# Intermittent Torsion of a Wandering Spleen: A Case of Recurrent Acute Abdominal Pain

**DOI:** 10.7759/cureus.110126

**Published:** 2026-06-02

**Authors:** Zahra Altaif, Maisa Altaif

**Affiliations:** 1 General Practice, Salmaniya Medical Complex, Manama, BHR; 2 Rheumatology, Salmaniya Medical Complex, Manama, BHR

**Keywords:** acute abdomen, case report, computed tomography, ectopic spleen, intermittent torsion, recurrent abdominal pain, splenectomy, splenic torsion, splenopexy, wandering spleen

## Abstract

Wandering spleen is a rare condition resulting from congenital or acquired laxity of the splenic ligaments, predisposing the organ to abnormal mobility and torsion of its vascular pedicle. We report a case of a patient presenting with recurrent episodes of acute abdominal pain due to intermittent torsion of a wandering spleen, characterized by spontaneous resolution between attacks and diagnostic uncertainty on initial evaluation. Clinical presentation was nonspecific, with episodic left-sided abdominal pain and variable examination findings, while routine laboratory investigations were largely unremarkable. Cross-sectional imaging played a decisive role in diagnosis, demonstrating ectopic splenic location in the lower abdomen with an elongated vascular pedicle and features consistent with intermittent torsion. The patient underwent surgical management with intraoperative confirmation of splenic mobility and vascular compromise without established infarction. Definitive treatment resulted in the resolution of symptoms and no recurrence on follow-up. This case highlights the importance of considering wandering spleen in the differential diagnosis of recurrent acute abdomen, particularly when imaging reveals a mobile or ectopic spleen. Early recognition through appropriate imaging is essential to prevent complications such as splenic infarction and to allow timely surgical intervention.

## Introduction

Wandering spleen is a rare clinical entity characterized by abnormal mobility of the spleen due to congenital absence, laxity, or elongation of its suspensory ligaments, including the gastrosplenic, splenorenal, and phrenicosplenic ligaments [1,2]. This anatomic variation allows the spleen to migrate from its normal left upper quadrant position to other regions of the abdomen or pelvis, where it may present as an incidental finding or with acute, subacute, or chronic symptoms [3,4]. Although it can occur at any age, it is most commonly reported in children and women of reproductive age, with proposed associations including congenital mesenteric defects, hormonal influences, and acquired ligamentous laxity [1,4]. Clinical presentation is highly variable, ranging from an asymptomatic abdominal mass to an acute abdomen due to torsion of the splenic pedicle, which can compromise vascular inflow and outflow [2,3].

Intermittent torsion of wandering spleen represents a particularly challenging diagnostic scenario due to its nonspecific and episodic symptom pattern [4,5]. Recurrent abdominal pain may mimic more common conditions, such as renal colic, appendicitis, gynecological emergencies, or functional gastrointestinal disorders, often leading to delayed diagnosis [3-6]. Cross-sectional imaging, particularly contrast-enhanced computed tomography, plays a central role in diagnosis by demonstrating ectopic splenic location, elongated vascular pedicle, and the characteristic “whirl sign” of torsion [1,5]. Early recognition is critical, as delayed diagnosis may result in splenic infarction, abscess formation, or life-threatening complications [1-4]. Management is typically surgical, ranging from splenopexy in viable spleens to splenectomy in cases of infarction or non-viability [2,3].

## Case presentation

The patient was a young adult presenting with recurrent episodes of acute, severe abdominal pain over a period of several months. The pain was characteristically sudden in onset, colicky in nature, and predominantly localized to the left upper quadrant with intermittent radiation to the periumbilical region and lower abdomen. Episodes were variably associated with nausea and occasional non-bilious vomiting, with spontaneous partial resolution over hours to days. Between episodes, the patient reported vague abdominal discomfort and a sensation of fullness, but no persistent pain, fever, altered bowel habits, gastrointestinal bleeding, or weight loss. There was no history of prior abdominal surgery, trauma, known hematologic disease, or chronic liver disease. Past medical history was otherwise unremarkable, and there was no family history of connective tissue disorders or similar abdominal complaints.

On presentation during the most recent acute episode, the patient appeared distressed and was intermittently writhing due to pain. Vital signs demonstrated mild tachycardia but were otherwise hemodynamically stable, with no fever or hypotension. Abdominal examination revealed a palpable, mobile, tender mass in the left lower and mid-abdominal region, with maximal tenderness elicited on deep palpation. The mass was difficult to clearly delineate due to guarding, but it appeared to change position with patient movement. There was no generalized peritonism, rebound tenderness, or signs of acute surgical abdomen. Bowel sounds were present and normal. Examination of other systems, including cardiopulmonary and neurological assessments, was unremarkable. No stigmata of chronic liver disease, lymphadenopathy, or splenomegaly in the usual anatomical location were identified.

Initial laboratory investigations, including complete blood count, C-reactive protein, liver function tests, serum amylase and lipase, renal profile, and coagulation studies, were largely within normal limits. Mild leukocytosis was noted during painful episodes, which normalized during asymptomatic intervals. Lactate levels remained within normal range, and there was no biochemical evidence of pancreatitis or hepatobiliary pathology. Urinalysis was unremarkable. Given the recurrent nature of symptoms and unclear clinical localization, cross-sectional imaging was pursued for further evaluation (Table 1).

**Table 1:** Laboratory investigations at presentation during an episode of acute abdominal pain Mild inflammatory changes were noted, including leukocytosis and slightly elevated C-reactive protein, while all other hematological, biochemical, liver, pancreatic, renal, and metabolic parameters remained within normal limits. Lactate levels were normal, with no biochemical evidence of tissue ischemia, pancreatitis, hepatobiliary dysfunction, or urinary tract pathology.

Parameter	Result	Reference Range
Hemoglobin (g/dL)	13.8	12.0–16.0
White blood cell count (×10⁹/L)	11.6	4.0–10.0
Neutrophils (%)	78	40–75
Platelet count (×10⁹/L)	265	150–400
C-reactive protein (mg/L)	6	<5
Serum sodium (mmol/L)	139	135–145
Serum potassium (mmol/L)	4.2	3.5–5.1
Urea (mmol/L)	4.8	2.5–7.8
Creatinine (µmol/L)	72	60–110
Total bilirubin (µmol/L)	10	3–21
Alanine aminotransferase (U/L)	22	0–40
Aspartate aminotransferase (U/L)	19	0–40
Alkaline phosphatase (U/L)	88	44–147
Serum amylase (U/L)	54	30–110
Serum lipase (U/L)	32	13–60
Lactate (mmol/L)	1.3	0.5–2.2

Ultrasound examination of the abdomen demonstrated the absence of the spleen in its usual left upper quadrant position, with a heterogeneous soft tissue mass identified in the lower abdomen/pelvic region, raising suspicion of an ectopic or wandering spleen. Doppler interrogation suggested variable and intermittently reduced splenic vascular flow, although assessment was limited by patient discomfort and bowel gas (Figure 1). Contrast-enhanced computed tomography (CT) of the abdomen and pelvis was subsequently performed, including imaging during symptomatic presentation. CT demonstrated a markedly displaced spleen located in the mid to lower abdomen, connected to an elongated and lax vascular pedicle. The spleen exhibited intermittent changes in orientation consistent with torsion and detorsion, with associated congestion and mild heterogeneous enhancement. At times of symptom exacerbation, there was evidence of twisting of the splenic vascular pedicle producing a “whirl sign,” with transient compromise of venous outflow more pronounced than arterial inflow. No evidence of splenic infarction was identified, although transient perfusion heterogeneity was noted. There was no associated bowel obstruction, free intraperitoneal air, or other acute intra-abdominal pathology (Figure 2).

**Figure 1:**
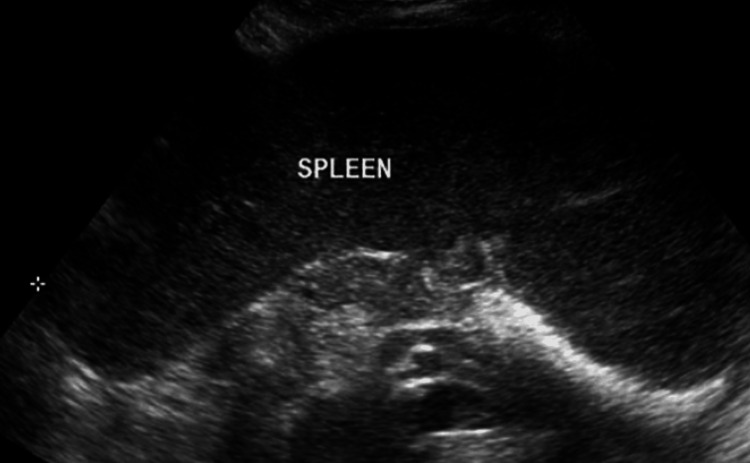
Ultrasound demonstrating ectopic splenic location in a patient with wandering spleen Transabdominal ultrasound image showing absence of the spleen from its normal left upper quadrant position with identification of an ovoid, heterogeneous soft tissue structure in the lower abdomen consistent with an ectopic spleen. The findings raise suspicion for wandering spleen, particularly in the context of recurrent abdominal pain.

**Figure 2:**
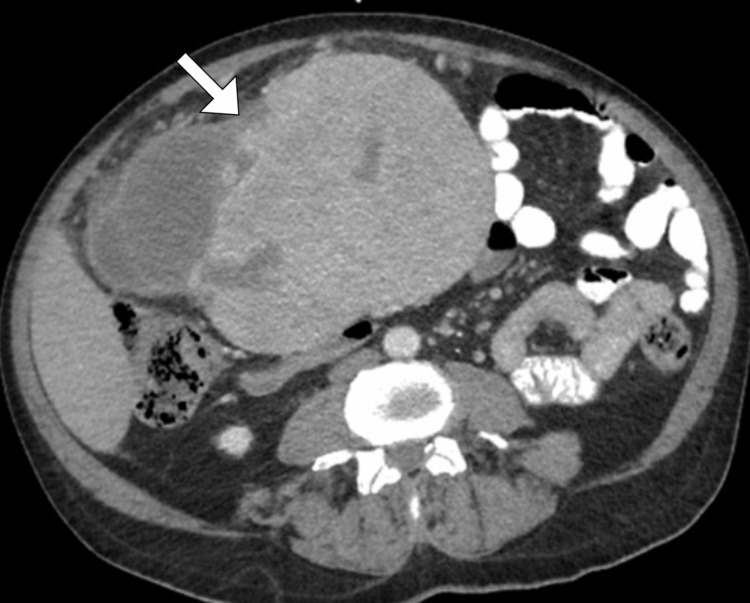
Contrast-enhanced CT demonstrating ectopic spleen with cystic changes indicative of infarcts Axial coronal contrast-enhanced computed tomography image of the abdomen and pelvis demonstrating the presence of an ectopic spleen located in the mid to lower abdomen. The spleen is heterogeneous in enhancement, with areas of cystic/necrotic changes indicative of an infarct in the setting of torsion.

The patient was admitted under general surgery for definitive management. Initial management consisted of intravenous fluid resuscitation, analgesia, and close monitoring for signs of splenic infarction or peritonitis. Given the recurrent nature of torsion episodes and the risk of progression to infarction, surgical intervention was planned. Intraoperatively, a markedly mobile spleen was identified, attached by an elongated and attenuated splenic pedicle with absent or lax supporting ligaments. The spleen was found to undergo partial torsion with evidence of venous congestion but preserved arterial inflow, consistent with intermittent torsion rather than complete infarction. After careful detorsion and assessment of splenic viability, the decision was made based on intraoperative findings and institutional protocol to proceed with definitive management.

The postoperative course was uneventful, with resolution of abdominal pain and gradual normalization of inflammatory markers. Oral intake was reintroduced and well-tolerated, and the patient was discharged in stable condition with planned outpatient follow-up. Shortly after surgery, the patient developed a headache and eye discomfort, which prompted ophthalmology assessment, including baseline optical coherence tomography and automated visual field testing performed at an external ophthalmology center. These investigations were obtained to document visual status and exclude concurrent neuro-ophthalmic pathology. The patient’s symptoms subsequently improved, with no progression or emergence of additional neurological or visual deficits. At follow-up, the patient reported complete resolution of abdominal symptoms with no recurrence. 

## Discussion

Wandering spleen with intermittent torsion is an uncommon but clinically significant cause of recurrent abdominal pain and acute abdomen, with fewer than a few hundred cases reported in the literature [6-10]. The condition arises from congenital maldevelopment or acquired laxity of the splenic suspensory ligaments, resulting in excessive splenic mobility and susceptibility to torsion of the vascular pedicle [2,6]. In the present case, the patient’s recurrent, self-limiting episodes of left-sided abdominal pain reflect intermittent torsion and spontaneous detorsion, a pathophysiological mechanism that explains both the episodic symptomatology and the diagnostic delay frequently encountered in such patients [1,4,6]. This intermittent vascular compromise can lead to venous congestion, splenic enlargement, and transient ischemia, while complete torsion may progress to infarction if not promptly recognized and managed [2,5,7].

The diagnostic challenge in wandering spleen lies in its rarity and nonspecific clinical presentation, which often overlaps with more prevalent causes of acute abdominal pain. In this case, initial differential diagnoses appropriately included renal colic, gastrointestinal inflammatory conditions, and gynecologic emergencies, reflecting the broad clinical spectrum that must be considered in patients with recurrent abdominal pain [1,3,5]. Physical examination may reveal a mobile abdominal mass with the absence of splenic dullness in the left upper quadrant, but these findings are often subtle or overlooked. Laboratory studies are generally non-contributory unless complications such as infarction or hypersplenism develop [2,4]. Therefore, imaging plays a pivotal role in diagnosis. Ultrasound may suggest ectopic splenic location; however, contrast-enhanced computed tomography remains the gold standard, demonstrating absence of the spleen from its normal position, an ectopic abdominal or pelvic spleen, and a characteristic twisted vascular pedicle known as the “whirl sign” [10-12].

Management strategies for wandering spleen are guided primarily by splenic viability at the time of diagnosis. Splenopexy is generally preferred in cases where the spleen is viable, particularly in pediatric and young adult populations, in order to preserve immunological function [5-8]. Various surgical techniques have been described, including mesh splenopexy and laparoscopic fixation, both of which aim to prevent recurrent torsion while maintaining splenic tissue. In contrast, splenectomy is reserved for cases complicated by infarction, hypersplenism, or non-viable splenic tissue [3,6]. In the current case, intraoperative findings of vascular congestion without established infarction supported an organ-preserving approach in principle; however, the final surgical decision is often influenced by intraoperative assessment, institutional expertise, and the risk of recurrent torsion if fixation is not deemed durable. This highlights the ongoing lack of consensus regarding optimal surgical management in borderline viability cases.

The importance of early recognition cannot be overstated, as delayed diagnosis increases the risk of irreversible splenic infarction, abscess formation, and septic complications, which carry significant morbidity [1,5]. Moreover, intermittent torsion represents a particularly deceptive phenotype, as spontaneous detorsion may temporarily relieve symptoms and normalize clinical findings, thereby masking the underlying pathology [2,5,7]. Awareness of this dynamic presentation is essential for clinicians and radiologists, particularly when evaluating unexplained recurrent abdominal pain with a mobile or ectopic abdominal mass [4-7]. From a radiological perspective, targeted assessment of splenic position and vascular pedicle integrity should be included in abdominal imaging protocols when suspicion arises, even in the absence of overt left upper quadrant pathology [2,4].

This case contributes to the limited body of literature describing intermittent torsion of wandering spleen and reinforces several key clinical principles. First, recurrent abdominal pain with a migratory or episodic pattern should prompt consideration of rare anatomical causes when routine investigations are unrevealing. Second, correlation of imaging with symptom timing can significantly enhance diagnostic yield in conditions characterized by intermittent vascular compromise. Third, definitive surgical management should be individualized, balancing splenic preservation against the risk of recurrent torsion and ischemic progression. Finally, increased awareness among emergency physicians, surgeons, and radiologists is essential to reduce diagnostic delay and improve outcomes. Given the rarity of the condition, further accumulation of case-based evidence and multicenter experience is necessary to refine management algorithms and establish clearer indications for splenopexy versus splenectomy in patients with viable but torsion-prone spleens.

## Conclusions

Wandering spleen with intermittent torsion is a rare but important and potentially life-threatening cause of recurrent abdominal pain, often presenting with nonspecific and episodic symptoms that can mimic more common abdominal and gynecological conditions. This case highlights the critical role of maintaining a high index of suspicion in patients with unexplained, recurrent, and migratory abdominal pain, particularly when physical examination reveals a mobile abdominal mass or when the spleen is not visualized in its normal anatomical position. Cross-sectional imaging, especially contrast-enhanced CT performed during symptomatic episodes, is essential for diagnosis and may demonstrate dynamic vascular compromise such as the characteristic whirl sign. Early recognition is crucial to prevent progression to splenic infarction and avoid delayed or inappropriate management. Definitive treatment should be individualized based on splenic viability, with splenopexy preferred when feasible to preserve immunological function. The key take-home message is that intermittent torsion of wandering spleen should be considered in the differential diagnosis of recurrent acute abdomen, as timely imaging and surgical intervention can be both organ-saving and potentially life-preserving.
